# Physiotherapy in lymphangioleiomyomatosis: a systematic review

**DOI:** 10.1080/07853890.2022.2128401

**Published:** 2022-10-10

**Authors:** Victoria Maria Garcia de Medeiros, Jéssica Gonçalves de Lima, Claudia Rosa, Juliana Rega, Mauro Felippe Felix Mediano, Luiz Fernando Rodrigues Junior

**Affiliations:** a Education and Research Department, National Institute of Cardiology, Rio de Janeiro, Brazil; b Physiotherapy Service, National Institute of Cardiology, Rio de Janeiro, Brazil; c Laboratory of Clinical Research on Chagas Disease, Evandro Chagas National Institute of Infectious Diseases, Oswaldo Cruz Foundation, Rio de Janeiro, Brazil; d Department of Physiological Sciences, Biomedical Institute, Federal University of the State of Rio de Janeiro, Rio de Janeiro, Brazil

**Keywords:** Lymphangioleiomyomatosis, physiotherapy, rehabilitation, exercise

## Abstract

**Background:**

Lymphangioleiomyomatosis (LAM) is associated with progressive dyspnoea and exercise intolerance, but despite the central role of physiotherapy on pulmonary rehabilitation, there is a huge lack of physiotherapy approaches used specifically for LAM patients.

**Objective:**

to identify the physiotherapeutic strategies used in the treatment of patients with LAM.

**Materials and methods:**

This is a systematic review of literature. Searches were performed (in PubMed, Lilacs, Embase and PEDro databases) with the keywords “Lymphangioleiomyomatosis” and “Physiotherapy,” and its variations. Articles describing physiotherapy interventions were included in the study. Data extracted from the studies were authors, year, country of publication, sample size, physiotherapy intervention, time/frequency/duration of intervention protocols, instruments used to measure results and main findings. Methodological quality of studies was evaluated by PEDro Scale (clinical trials), Newcastle–Ottawa Scale (NOS; observational studies) and CARE checklist (case reports), respectively.

**Results:**

A total of 82 articles identified, three duplicates were removed, 71 studies were excluded after title and abstract reading and four after full-text reading, all due to absence of association with the study topic. Four studies were included in the present review. Cardiorespiratory physiotherapy with endurance and resistance training were identified as physiotherapeutic strategies to improve lung function, functional capacity, depression symptoms and quality of life in LAM.

**Conclusions:**

Endurance and resistance training is the keystone for physiotherapy in patients with LAM, but despite the reported benefits, there is a huge lack of studies related to the modalities, safety and dosage of physiotherapy prescription for patients with LAM.

**KEY MESSAGES**
- Lymphangioleiomyomatosis (LAM), a rare disease, leads to progressive dyspnoea and exercise intolerance;
- Physiotherapy can improve dyspnoea and exercise intolerance in LAM through endurance and resistance exercises.

## Introduction

Lymphangioleiomyomatosis (LAM) – a rare low-grade neoplasia that almost exclusively affects women at childbearing age – is characterized by cystic formation, predominantly in lungs, lymphatics and kidneys, with typical clinical manifestations of progressive dyspnoea, recurrent pneumothorax, pleural effusion and exercise intolerance [1–4]. LAM may occurs in patients with tuberous sclerosis complex (TSC), an autosomal-dominant disorder caused by mutations in the TSC1 or *TSC2* genes, characterized by mental retardation, autism, seizures and hamartomatous lesions in the brain, heart, skin, kidney, eyes, lungs and liver. Also, noninherited form of LAM (sporadic LAM) is caused by somatic mutations of the *TSC2* gene. The estimated LAM prevalence is approximately 3.3–7.7 per 1,000,000 women [3].

Clinical manifestation of LAM may remain stable for prolonged periods or evolve with rapid and progressive symptoms and impairment of pulmonary function [4]. Thus, as soon as individuals progress with severely impaired lung function (New York Heart Association [NYHA] functional class III or IV), extremely compromised exercise capacity and need for oxygen therapy, the Brazilian Society of Pulmonology and Tisiology recommend lung transplantation, since therapeutic options are limited [5].

In this context, it is important to monitor the decline in lung function through imaging and pulmonary functional tests (PFTs), monitoring the reduction of forced expiratory volume in one second (FEV1) and carbon monoxide diffusing capacity (DLCO), in addition to decline in functional capacity, assessed by the physical therapist through functional tests, such as the 6-minute walk test (6MWT) [3, 5]. In addition, functional performance should be monitored during exercise, either by maximal oxygen consumption (VO_2max_) obtained during a cardiopulmonary exercise test (CPET) or by the 6MWT [3].

Non-pharmacological clinical strategies, such as cardiorespiratory physiotherapy, already widely indicated for patients with chronic lung diseases, can contribute reducing dyspnoea, increasing exercise tolerance, improving lung function and quality of life in patients with LAM [6, 7]. However, although the European Respiratory Society [7] mentions the pulmonary rehabilitation for the management of dyspnoea in LAM, the literature is scarce regarding to the physiotherapy approaches used specifically for LAM patients. Therefore, the objective of this study was to identify physiotherapeutic strategies used in the treatment of patients with LAM.

## Materials and methods

This systematic review followed the recommendations of the Preferred Reports Reviews for Systematic Reviews and Meta-Analyses [8], as well as the Tutorial for Writing Systematic Reviews [9]. The review protocol was published in the PROSPERO registry database: CRD42021273260.

### Search strategy

For the formulation of the clinical question of this systematic review we used the Population, Intervention, Outcome (PIO framework – a variation of PICO framework without control stage), considering as population: LAM; as intervention: physiotherapy; and as outcomes: physical therapy modalities that are being used (primary outcome) and rehabilitation effectiveness/exercise tolerance/physical conditioning/functional independence/quality of life/mortality (secondary outcomes).

Bibliographic searches were performed in PubMed, Lilacs, Embase and PEDro databases, without year and language restrictions. The searches were realized in October 2021 using the descriptors and synonyms as follows: “Lymphangiomyomatosis” (OR “Lymphophangioleiomyomatosis” OR “Lymphophangiomyomatosis” OR “lymphangiophangiomyomatosis” OR “Lymphangiomyomatosis”) and “Physiotherapy” (OR “Physiotherapy” OR “Rehabilitation” OR “Exercise” OR “Pulmonary Rehabilitation”). The complete search strategy can be found in (https://osf.io/k6qzw/). These terms were based on a search in the Medical Subject Headings (MeSH) list of PUBMED and Health Sciences Descriptors (DeCS) of the Virtual Health Library. Initially, the main researcher performed the search in all databases using the Covidence systematic review software (Veritas Health Innovation, Melbourne, Australia, available at www.covidence.org), identified and eliminated duplicate articles. Then, two different researchers read the titles and abstracts of all remaining articles, excluding those not related to the topic of the review. Subsequently, two other researchers completed the reading of the full text of the selected articles, realized data extraction and evaluated methodological quality.

### Eligibility criteria

Articles published in any language and year, describing physical therapy interventions in LAM patients were selected for the review and were included in the study. Abstract of congress or proceedings of events were excluded from the study.

### Data extraction

Two reviewers extracted data independently, being blinded to the other research decisions. Disagreements were solved by the main researcher. The data extracted from the studies were authors, year of publication, country of origin, sample size, physical therapy intervention, time/frequency/duration of intervention protocols, instruments used to measure results and main findings (outcomes).

### Analysis of the methodological quality of the studies

The methodological quality of the studies was assessed according to the type of study. Clinical Trials were evaluated using the PEDro Scale, which is a valid and reliable instrument composed of 11 items that analyse the methodological design and evaluate the description of inclusion and exclusion criteria, blinding of evaluators, therapists and patients, the allocation of subjects, similarity of prognostic indicators between groups, assessment of key outcomes in at least 85% of subjects randomized between groups, analysis of key outcomes by “intent to treat” when it was not possible to receive the treatment or the condition control by allocation, and the statistical description of intergroup differences or measures of variability for at least one key outcome [10].

Observational studies were evaluated using the Newcastle–Ottawa Scale (NOS), which is a specific instrument for case control and cohort studies, consisting of three categories and eight items: selection (4 items), comparability (1 item) and outcome (3 items). A study can be awarded a maximum of one star (*) for each numbered item within the categories. A maximum of two stars can be given to each item in the comparability category. Each star represents 1 point, with the maximum scale score being 9 points [11].

The case reports were evaluated using CARE checklist that have been developed by an international group of experts to support an increase in the accuracy, transparency and usefulness of case reports [12].

## Results

**Figure 1.**
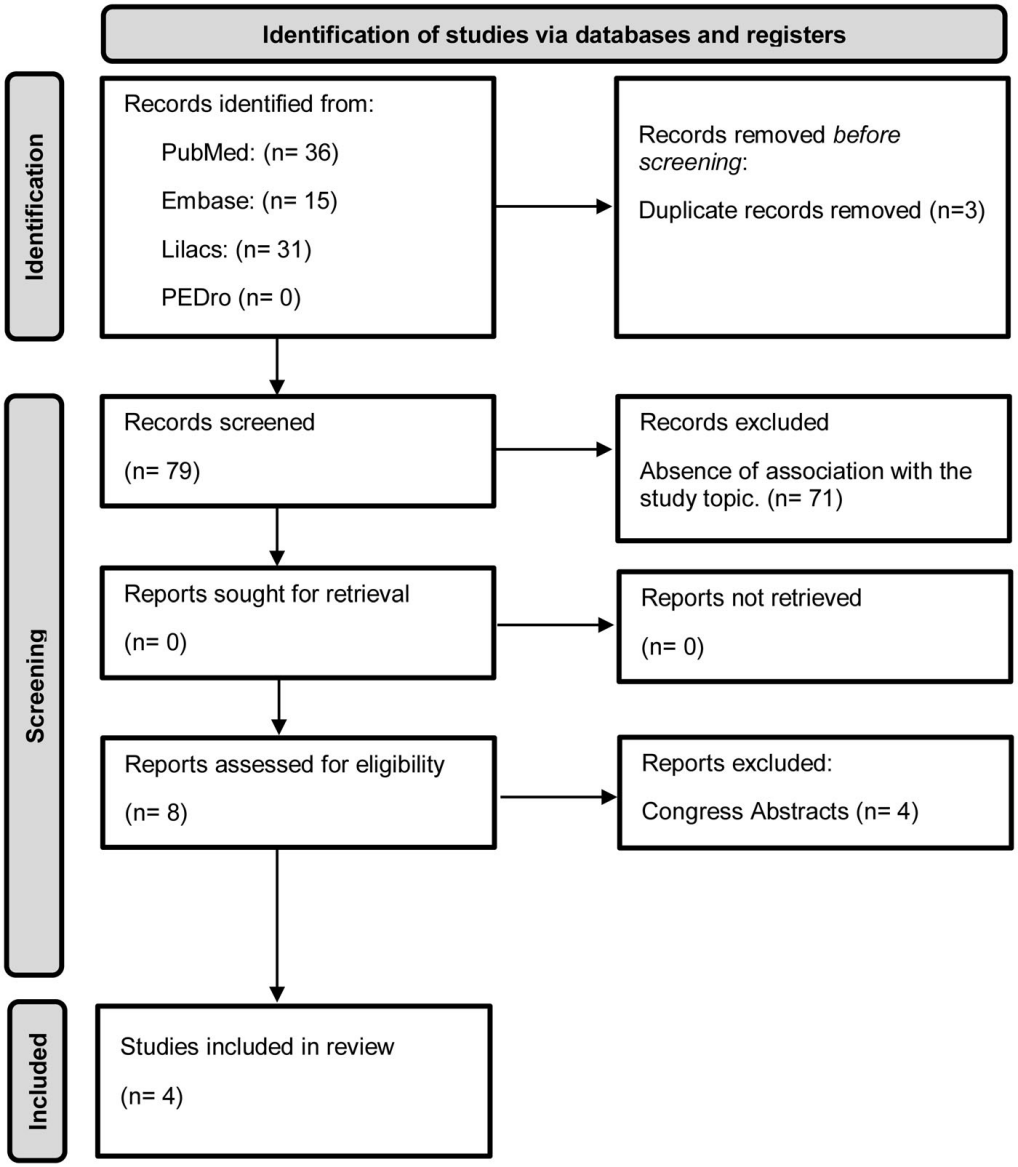
Study flowchart.

**Figure 2.**
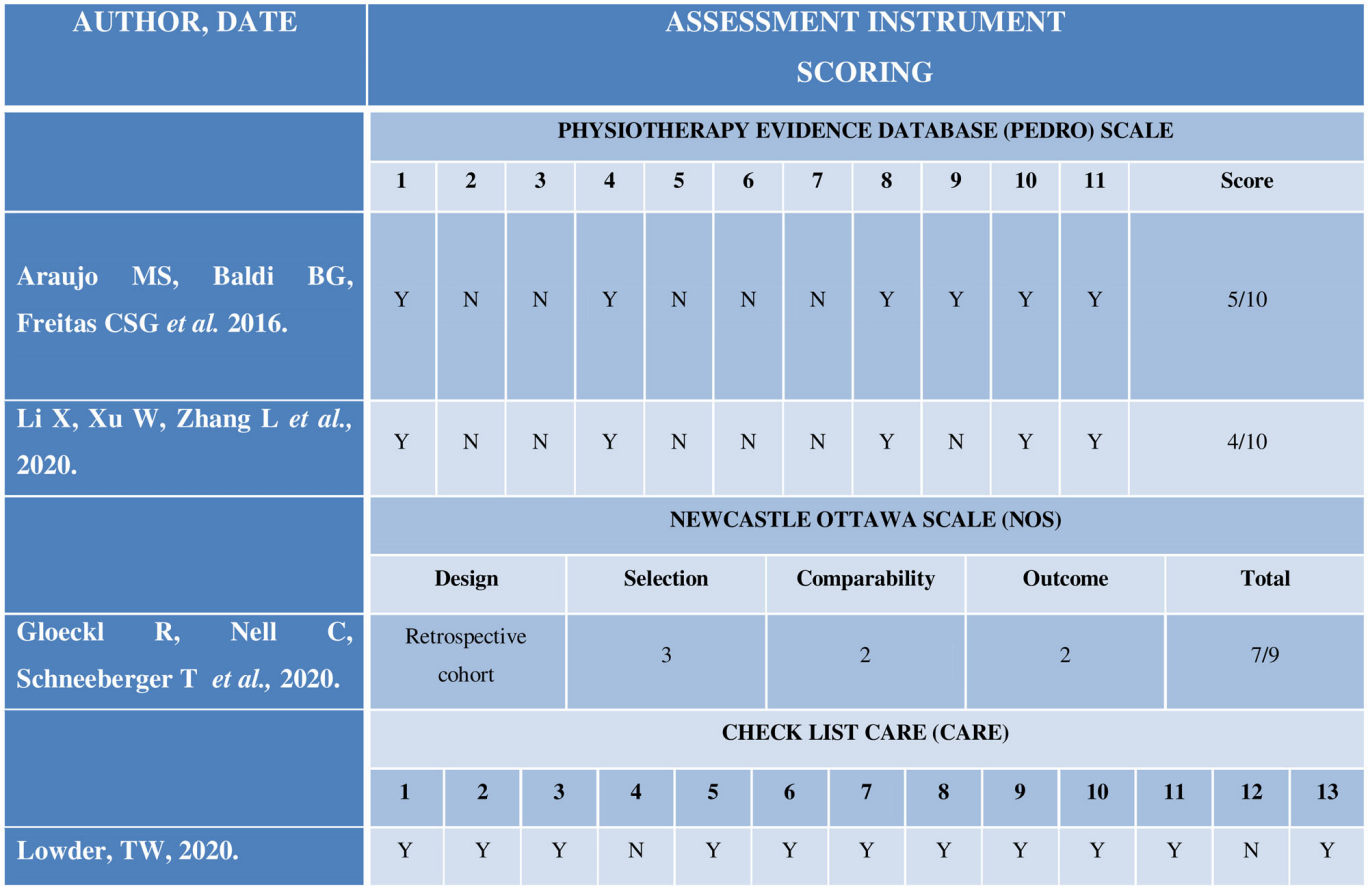
Methodological quality assessment of the studies included in the review.

A total of 82 articles were selected, from those three duplicates were removed and 71 were excluded after title and abstract reading due to the absence of association with the study topic. The remaining eight were read in full text for screening for eligibility, four of them were excluded for the absence of association with the study topic. So, four studies were included in this review (Figure 1), one case report (fulfilling 11 of the 13 items on CARE checklist, which demonstrates an adequate design for a case report), two non-randomized clinical trials (score ranging between 4 and 5 scores on the PEDro scale, which demonstrates a low methodological quality) and one observational study (score of 7 according to the NOS, which demonstrates good methodological quality), and analysed based on the score on the Physiotherapy Evidence Database scale (Table 1), NOS and CARE checklist (Figure 2).

**Table 1.** Main outcomes of the study.

Author, year. country	Study design	Disease severity	Sample size	Age/gender	Physiotherapy interventions	Duration, Number of sessions	Control group	Assessment	Main outcomes
Araújo et al. [14]. Brazil.	Non-randomized, clinical Trial.	6MWT (m): 517 FEV1 (L): 2.04 ± 0.84 FEV1%pred: 72 ± 28 FEV1/FVC: 0.75 (0.57–0.8) VO_2peak_ (mL/kg/min): 16.4 ± 49	40	43 ± 10 years old/women.	Endurance and resistance training and education instructions. Aerobic training was performed at a heart rate corresponding to two-thirds of the difference between the anaerobic threshold (AT) and the respiratory compensation point, determined during an incremental cardiopulmonary exercise test (CPET). Oxygen supplementation was provided when necessary to maintain SpO_2_ above 90%.	24 sessions, 2×/weeks (60 min/session; 30 min of endurance and 30 min of resistance training).	Received only educational instructions and with the advertisement to maintain their physical activity routine.	PFT, CPET, 6MWT, CWRT, 1-RM test, mMRC, TDI; SGRQ; HADS.	The intervention improved exercise capacity (increasing endurance time during CWRT by 44% and distance walked on 6MWT by 59 m). The intervention improved peak VO_2_, daily physical activity, health-related quality of life, and muscle strength. It also reduced dyspnoea (39% of patients in the intervention group had a decrease in dyspnoea score (mMRC) when compared to the control group). The intervention group improved depression symptoms. No difference was observed in anxiety levels. There was no significant improvement in lung function after rehabilitation when compared to the control group.
Lowder, TW [13]. United States of America.	Case Report.	FEV1 (L): 2.53 FEV1/FVC (%): 66.3 FVC (L): 3.82 Peak Flow (L): 3.71 VO_2max_ (mL/kg/min): 29.43 DXA whole body (g/cm^2^): 1.3; DXA lumbar spine (g/cm^2^): 1.326.	1	29 years old/woman.	High-intensity aerobic (primarily treadmill/track running/sprinting) and resistance training.	1 year, 2×/weeks (60 min/session)	n/a	Graded exercise test (VO_2max_), PFT and bone mineral density	The intervention improved lung function (by increasing FEV1 – by 9%, FEV1/FVC – by 7% and peak flow –by 47%). Exercise tolerance increased by 20% (VO_2max_). Bone mineral density, FVC and body weigh remained steady along the intervention.
Li et al. [15]. China.	Non-randomized, clinical Trial.	FEV1 (mL): 2009 ± 639 FEV1%pred: 72.0 ± 21.6 FEV1/FVC (%): 62.6 ± 14.3 FVC (mL): 3196 ± 628 FVC%pred: 98.5 ± 16.5 VO_2peak_ (mL/kg/min): 15.4 ± 3.3	26	39.8 ± 8.5 years old for yoga group and 43.4 ± 9.2 years old for control group. Gender not mentioned.	Traditional hatha yoga; Patients realized yoga sessions consisting of yoga in asanas (postures) interspersed with chanting and pranayama (timed breathing).	24 weeks, 1×/week (90 min/session); Home exercise orientation 2×/weeks (15 min/session);	No intervention.	PFT (FEV1, VFC.), Incremental CPET (VO_2peak_ , AT); Daily activity assessment; 6MWT; Borg scale; HADS; SGRQ.	The intervention improved the distance walked on the 6MWT (18 ± 49 m in the control group *vs*. 55 ± 29 m in intervention group). No difference was found in the change in VO_2peak_ or PFT variables. The intervention group had an increase in AT, which may suggest an improvement in aerobic capacity. There was no significant difference in anxiety and depression levels between the yoga and control group.
Gloeckl et al. [6]. Germany	Retrospective analysis.	Advanced LAM. FEV 1 (L): 1.32 ± 0.74; FEV1 (%): 45.8 ± 24. IVC %pred: 72.1 ± 24.9 IVC (L): 2.5 ± 1.0 [2.3]	58	48.2 ± 10.3 [48.4] years old/woman	Multidisciplinary pulmonary rehabilitation programme with specialized content for patients with chronic respiratory diseases (including endurance and strength training). Patients also participated in structured general education sessions (disease management or oxygen 1: therapy) and respiratory physiotherapy, smoking cessation, nutritional and psychological counselling were provided on a case-by-case basis.	4 weeks/ 5–6 d/weeks consisted of daily exercise training sessions for 60 min.	–	PFT, 6MTW); Quality of Life (SF-36);	There were significant and clinically relevant improvements in exercise performance and quality of life after the pulmonary rehabilitation programme (PR). After RP, the 6MWD increased by 49 ± 50 m.

VO_2max_: maximal oxygen consumption; FVC: forced vital capacity; FEV1: forced expiratory volume during first second of forced expiration; PFT: pulmonary function test; VO_2_peak: peak oxygen consumption; FEV1%pred: percentage of predicted forced expiratory volume during first second of forced expiration; 6MWT: 6-minute walking test; CPET: cardiopulmonary exercise test; CWRT: constant work rate test; mMRC: modified Medical Research Council dyspnoea scale; TDI: Transitional Dyspnoea Index; FVC%pred: percentage of predicted forced vital capacity; SGRQ: St. George Respiratory Questionnaire; HADS: Anxiety and Depression Scale; AT: anaerobic threshold; SF-36: short-form 36 question health survey; AT: anaerobic threshold.

Table 1 presents the results of the manuscripts after data extraction. In the case report by Lowder [13], it was found that a high-intensity aerobic (mainly using the treadmill) and supervised resistance training during 1 year (2 times a week, 60 min per session) was able to improve lung function and exercise tolerance, while bone mineral density (BMD), forced vital capacity (FVC) and body weight remained stable. The assessments used were the PFT, graded exercise test (GTX) to measure VO_2max_ and an assessment of BMD, which were performed before starting the programme and every 3 months.

In the non-randomized clinical trial conducted by Araujo et al. [14], 27 women were allocated in intervention group (that performed a total of 24 exercise sessions performed twice a week, constituting 30 min of aerobic training and 30 min of strength training) and control group (that received only educational guidance and instruction not to change their physical activity routine). After the intervention, there was an improvement in exercise capacity assessed by constant work exercise test (CWRT) and 6MWT, in peak VO_2_ assessed by CPET, in performance of activities of daily living and quality of life, assessed by Saint George’s ¬ Respiratory Questionnaire (SGRQ). In addition, those in the intervention group reported improvements in symptoms of depression, assessed using the Hospital Anxiety and Depression Scale (HADS), and in the subjective sensation of exertion, assessed using the modifies Medical Research Council dyspnoea scale (mMRC).

In the non-randomized clinical trial conducted by Li et al. [15], the effects of Yoga on improving aerobic capacity, anxiety and depression levels in women with LAM were evaluated. The Yoga group held traditional Hatha Yoga sessions, composed by asanas (postures) and pranayamas (timed breaths), once a week, 90 min per session, in addition to home-based exercises, twice a week, 15 min per session, during 24 weeks of the intervention. The control group did not perform any intervention. An improvement in the 6MWT distance and an increase in the anaerobic threshold (AT) were observed. There was no significant difference in anxiety and depression levels between the Yoga and control groups.

Finally, Gloeckl et al. [6] retrospectively investigated 58 women with LAM that participated of a multidisciplinary pulmonary rehabilitation programe consisting of aerobic and strength exercises, performed 5–6 times a week, 60 min per session, during 4 weeks and, health education sessions, originally developed for patients with COPD. Despite not being specific for individuals with LAM, the programme demonstrated being effective in improving exercise performance (assessed by 6MWT) and quality of life (assessed by SF-36) [13].

## Discussion

In the studies included in this review, aerobic [6,13,14] and the strength training [6,14] were the most used physiotherapy techniques in LAM patients. Both, according to the literature [6,16], are commonly used as physiotherapy modalities in the rehabilitation of individuals with pulmonary disease and are considered low cost and easy to implement. Although there is no cardiopulmonary rehabilitation guideline in LAM, the European Respiratory Society [7] states that the benefits of rehabilitation in COPD can be extrapolated to LAM.

In the study by Li et al. [15], the practice of yoga was used as a rehabilitation strategy, through postures (asanas) and controlled breathing (pranayamas). Although yoga is uncommon as a cardiopulmonary rehabilitation tool, studies with COPD individuals [17,18] report improvement in exercise tolerance, pulmonary function parameters and quality of life, corroborating the findings of Li et al. [15] who mention an increase in FEV1 and in the distance from the 6MWT in the intervention group. The hypothesis is that pranayamas resemble two techniques that are commonly taught during pulmonary rehabilitation: diaphragmatic breathing and lip flexing.

The most used assessment methods were the PFT and the 6MWT, in addition to scales to assess depression, anxiety and quality of life, with the main ones being the Saint SGRQ and the HADS. Regarding the 6MWT, the American Thoracic Society (ATS) [19] recommends its use in patients with moderate to severe heart or lung disease, with the aim of providing a global assessment of exercise response and functional capacity.

In this review, three studies used the 6MWT to assess exercise tolerance, while the respiratory function was evaluated through the PFT by the variables vital capacity (VC), total lung capacity (TLC), FVC, FEV1 and the FEV1/FVC ratio. In addition, some of the studies also used the CPET and the CWRT.

CPET is an important method of evaluating aerobic performance, which subjects the individual to an exercise of increasing intensity until exhaustion or the appearance of symptoms, in order to measure ventilation (VE), oxygen consumption (VO_2_) and carbon dioxide (VCO_2_) production [20]. Similar to CPET, CWRT is highly responsive to therapeutic interventions and has become a way of studying exercise tolerance in individuals with COPD [21]. However, it is more sensitive and investigates a work rate domain most likely to be encountered in everyday life [21].

The evaluated studies identified that a cardiopulmonary rehabilitation programme for individuals with LAM, composed mainly of aerobic associated with strength training, 2–3 times a week, 60 min per session and lasting more than 4 weeks, is able to improve the lung function, exercise tolerance, reduce symptoms of dyspnoea [6,10–12], and improve depression and quality of life [10–12]. A recent systematic review with meta-analysis of pulmonary rehabilitation in individuals with COPD showed that a programme with intervention lasting from 4 to 12 weeks, including aerobic training and lower limb strengthening as standard training, is able to improve exercise tolerance, dyspnoea and quality of life [22], corroborating this review findings, despite the lacking description of strength training strategy in the manuscripts included in our review.

Limitations of this review included the small number of studies and their heterogeneity, which is attributed to the fact that LAM is a rare disease, making difficult the conduction of randomized clinical trials.

## Conclusions

The analysed studies showed that a cardiopulmonary rehabilitation programme including aerobic and strength training presented as the most important physiotherapeutic strategy capable of improving lung function and exercise tolerance in individuals with LAM. Despite the benefits of physiotherapy reported in the studies, there is a huge lack of studies related to other modalities, safety and effectiveness of physiotherapy prescription for patients with LAM.

## Data Availability

The complete search strategy can be found in (https://osf.io/k6qzw/).
